# Diagnostic performance of D-dimer in predicting pulmonary embolism in tuberculous pleural effusion patients

**DOI:** 10.1186/s12890-021-01546-y

**Published:** 2021-05-22

**Authors:** Xiaoming Li, Yajing Qin, Wenjing Ye, Xi Chen, Dezhi Sun, Xuejun Guo, Wen Gu

**Affiliations:** 1 Department of Respiratory Medicine, Xinhua Hospital, Shanghai Jiaotong University School of Medicine, 1665, Kongjiang Road, Shanghai, 200092 China; 2 Department of Respiratory and Critical Care Medicine, Weifang Respiratory Disease Hospital, Weifang NO.2 People’s Hospital, 7, Yuanxiao Street, Weifang, 261041 Shandong Province China

**Keywords:** Tuberculous pleural effusion, D-dimer, Pulmonary embolism, Diagnosis

## Abstract

**Background:**

Tuberculous pleural effusion (TPE) patients usually have elevated D-dimer levels. The diagnostic performance of D-dimer in predicting pulmonary embolism (PE) in the TPE population is unclear. This study aimed to assess the diagnostic performance of D-dimer for PE in the TPE population and explore its potential mechanism.

**Methods:**

We retrospectively analysed patients who were admitted to Xinhua Hospital and Weifang Respiratory Disease Hospital with confirmed TPE between March 2014 and January 2020. D-dimer levels were compared between patients with and without PE. To test the diagnostic performance of D-dimer in predicting PE, receiver operating characteristic curve analysis was performed. Positive predictive value (PPV) and negative predictive value (NPV) were also reported. To explore the potential mechanism of PE in TPE, inflammatory biomarkers were compared between PE and non-PE patients.

**Results:**

This study included 248 patients (170 males and 78 females) aged 43 ± 20.6 years. Elevated D-dimer levels (≥ 0.5 mg/L) were detected in 186/248 (75%) patients. Of the 150 patients who underwent computed tomography pulmonary angiography, 29 were diagnosed with PE. Among the TPE population, the PE patients had significantly higher D-dimer levels than the non-PE patients (median, 1.06 mg/L vs. 0.84 mg/L, *P* < 0.05). The optimal cut-off value for D-dimer in predicting PE in TPE was 1.18 mg/L, with a sensitivity of 89.7% and a specificity of 77.8% (area under curve, 0.893; 95% confidence interval 0.839–0.947; *P* < 0.01). The PPV was 49.1%, while the NPV was 96.9% at a D-dimer cut-off of 1.18 mg/L for PE. PE patients had lower median WBC and interleukin (IL)-8 values (5.14 × 10^9^/L vs. 6.1 × 10^9^/L, *P* < 0.05; 30.2 pg/ml vs. 89.7 pg/ml, *P* < 0.05) but a higher median IL-2 receptor value (1964.8 pg/ml vs. 961.2 pg/ml, *P* < 0.01) than those in the non-PE patients.

**Conclusions:**

D-dimer is an objective biomarker for predicting PE in patients with TPE. A D-dimer cut-off of 1.18 mg/L in the TPE population may reduce unnecessary radiological tests due to its excellent sensitivity, specificity, and NPV for PE. The imbalance of prothrombotic and antithrombotic cytokines may partly be attributed to the formation of pulmonary emboli in patients with TPE.

## Background

Tuberculosis (TB) is one of the major causes of death from infectious diseases worldwide. Tuberculous pleural effusion (TPE) is one of the most common forms of extrapulmonary TB caused by *Mycobacterium* infection in the pleural space [1]. Its frequency varies according to the TB burden in different regions. As reported, it is less than 10% in low TB burden countries and over 40% in countries with high TB burden. TPE accounts for 6.5–8.7% of reported TB cases in China and is the most common cause of pleural effusion, especially in young adults [2].

Pulmonary embolism (PE) due to endogenous or exogenous thrombosis in the pulmonary arterial trunk or its branches is a potentially life-threatening disease [3]. Accurate and prompt diagnosis is essential for reducing mortality. D-dimer is formed when cross-linked fibrin breaks down. In patients who are suspected of having PE, plasma D-dimer levels correlate with the probability of having PE [4]. To increase the specificity of D-dimer testing and avoid unnecessary costly and potentially harmful computed tomography pulmonary angiography (CTPA) tests, the 2019 ESC Guidelines endorse using clinical pretest probability (C-PTP) together with D-dimer levels to rule out PE instead of a fixed cut-off level [5]. However, recent studies have suggested that strategies using clinical probability and D-dimer have limited diagnostic performance when PE is associated with complications. Goodacre et al. [6] reported that during pregnancy, the YEARS/D-dimer strategy had a sensitivity of 58.3% and a specificity of 44%, while the Geneva/D-dimer strategy had a sensitivity of 75% and a specificity of 20.8% for PE. The level of D-dimer is elevated in most patients with pleural effusion and is considered a useful biomarker for TPE diagnosis [7, 8]. It is well established that PE should be ruled out if the patients have a normal D-dimer level [9]. PE has similar symptoms, such as chest pain and shortness of breath, with TPE. In addition to elevated D-dimer levels, whether to perform CTPA to confirm the PE diagnosis is a dilemma for clinicians due to its high cost, time consumption, and potential risk of kidney injury.

To assess the diagnostic performance of D-dimer for PE in the TPE population and explore the potential mechanism of PE in TPE patients, we analysed the clinical data of TPE patients in our centres and made contributions to guide clinical decision-making.

## Methods

### Patients

We retrospectively analysed patients with confirmed TPE between March 2014 and January 2020 who were admitted to the Department of Respiratory Medicine, Xinhua Hospital affiliated to Shanghai Jiao Tong University School of Medicine and the Department of Respiratory and Critical Care Medicine, Weifang Respiratory Disease Hospital in China. Identification of *Mycobacterium tuberculosis* in pleural effusion was done by performing acid-fast staining, culture, or polymerase chain reaction and suggestive clinical and radiologic findings. Patients were excluded if they had conditions that may influence the D-dimer levels, such as an active tumour, hepatic insufficiency, renal insufficiency, and pregnancy.

This retrospective study only collected the clinical data of patients, did not interfere with the treatment plan of patients, and did not pose any risk to patients' physiology. We did our best to protect the information provided by patients without disclosing their personal privacy. All protocols were approved by the Ethics Committee of Xin Hua Hospital, affiliated with Shanghai Jiao Tong University School of Medicine. (NO. XHEC-D-2020-183). Written informed consent was obtained from all patients.

### Data collection

According to the medical record, data regarding patients’ characteristics—such as age, sex, previous medical history, current symptoms, D-dimer, C-reactive protein (CRP), white blood cell (WBC), and erythrocyte sedimentation rate (ESR) as well as interleukin (IL)-6, IL-8, TNF-α, and IL-2 receptor of the first blood test—were collected and analysed. Imaging results, including CTPA and compression ultrasound with Doppler, were collected.

### Statistical analyses

SPSS version 22.0 (IBM Corporation, New York) was used for the statistical analysis. The Mann–Whitney U test was used for nonparametric continuous variables. Receiver operating characteristic (ROC) curve analysis was used to evaluate the threshold value of D-dimer in differentiating PE from non-PE in patients with TPE. A cut-off point was determined as the value of the parameter that maximised the sum of specificity and sensitivity based on Youden’s index. Sensitivity and specificity were calculated to assess the diagnostic performance of D-dimer. PPVs and NPVs were also reported. Statistical significance was set at *P* < 0.05.

## Results

**Fig. 1:**
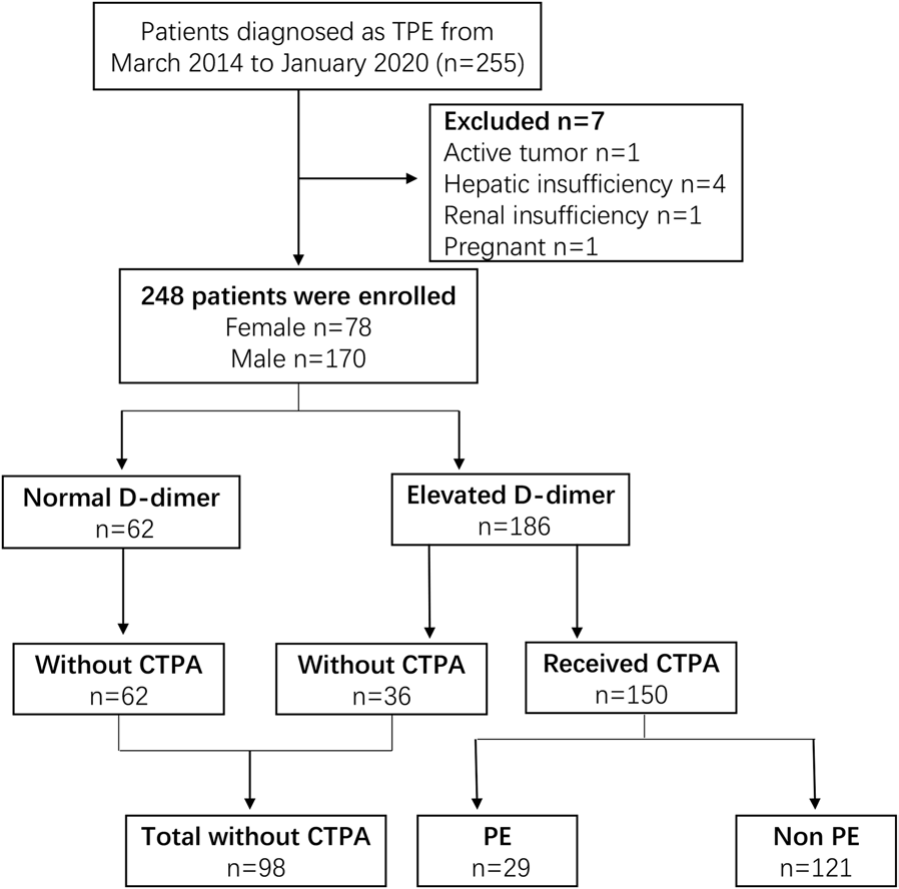
Flow chart of participants through the study. PE, pulmonary embolism; CTPA, computed tomography pulmonary angiogram

A total of 255 patients were finally diagnosed with TPE between March 2014 and January 2020 at our centres. We excluded seven patients (active tumour = 1, hepatic insufficiency = 4; renal insufficiency = 1, and pregnant = 1) with conditions that may influence D-dimer levels. A total of 248 patients (170 males and 78 females) aged 43 ± 20.6 years were finally included in this study. Elevated D-dimer levels (≥ 0.5 mg/L) were detected in 186/248 (75%) patients. Of 186 patients, 150 underwent CTPA, 29 were diagnosed with PE, and 121 patients had no PE on diagnostic imaging. The overall PE rate was 11.7% (29 of 248 patients) in the TPE population. A total of 98 patients did not undergo a CTPA, including 62 with normal D-dimer levels and 36 with elevated D-dimer levels. Figure 1 shows the flow of participants throughout the study, and Table 1 outlines the characteristics of the patients included in and excluded from the analysis.

**Table 1:** Characteristics of the study population (n = 255)

Characteristics	Count (%) or mean (SD) or median (range)	
	Include (n = 248)	Exclude (n = 7)
Male	170 (68.5%)	4 (57.1%)
Female	78 (31.5%)	3 (42.9%)
Age (years)	43 (20.6)	62.3 (16.1)
*Symptoms*		
Cough	185 (74.6%)	5 (71.4%)
Fever	128 (51.6%)	2 (28.6%)
Chest pain	126 (50.8%)	3 (42.9%)
Dyspnea	123 (49.6%)	6 (85.7%)
Expectoration	100 (40.3%)	3 (42.9%)
D-dimer (mg/L)	0.83 (0.17–5.02)	2.41 (1.68–5.76)

**Fig. 2:**
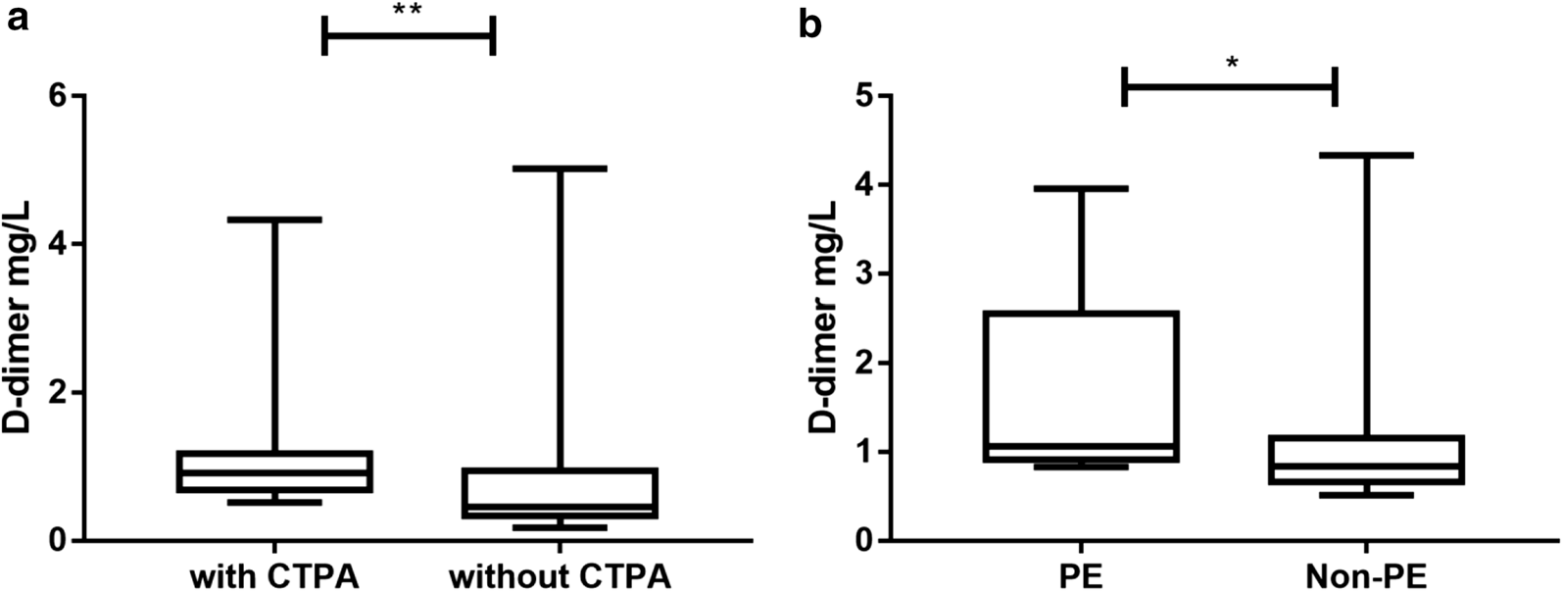
**A** D-dimer levels between patients who received and did not receive computed tomography pulmonary angiography (median, 0.92 mg/L vs. 0.46 mg/L, *P* < 0.01). **B** D-dimer levels between pulmonary embolism (PE) and non-PE among tuberculous pleural effusion patients (median, 1.06 mg/L vs 0.84 mg/L, *P* < 0.05)

**Fig. 3:**
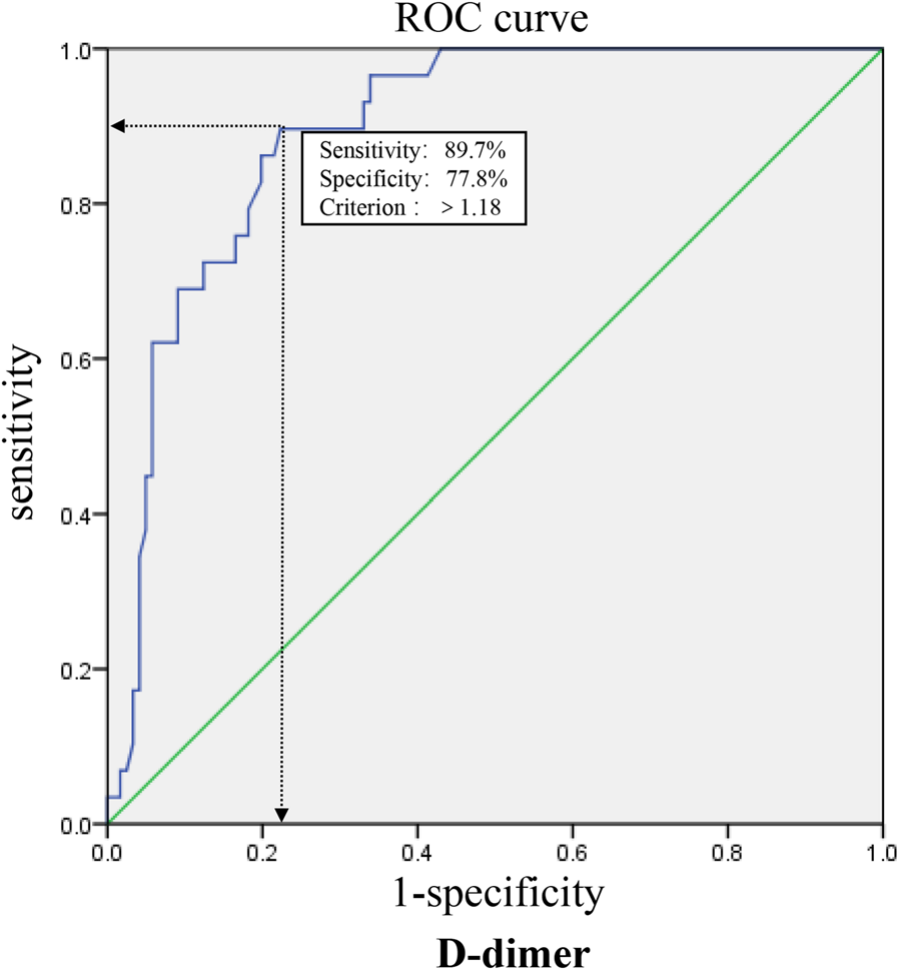
Receiver operating characteristic curves of D-dimer for differential diagnosis of pulmonary embolism (PE) (n = 29) versus non-PE (n = 121) among tuberculous pleural effusion patients. The area under the receiver operating characteristics curve was 0.893

In our study, the median D-dimer in patients who underwent CTPA was significantly higher than in those who did not undergo CTPA (0.92 mg/L vs. 0.46 mg/L, *P* < 0.01, Fig. 2A). Among the patients who received CTPA, the median D-dimer level in the PE patients was significantly higher than in the non-PE patients (1.06 mg/L vs. 0.84 mg/L, *P* < 0.05, Fig. 2B). To differentiate the PE group from the non-PE group, we calculated the ROC curve. The area under the ROC curve (AUC) was 0.893 (95% confidence interval 0.839–0.947; *P* < 0.01) (Fig. 3). The ROC curve analysis suggested that the best cut-off point for D-dimer in predicting PE in TPE was 1.18 mg/L, with a sensitivity of 89.7% and a specificity of 77.8%. The positive predictive value (PPV) was 49.1% (95% CI 35.3–63) while the negative predictive value (NPV) was 96.9% (95% CI 90.6–99.2) at a D-dimer cut-off of 1.18 mg/L for PE (Table 2). All 186 patients with elevated D-dimer levels received compression ultrasound of the lower extremity deep vein, however, none of them had deep thrombosis.

**Table 2:** Diagnosis performance of D-dimer > 1.18 mg/L in predicting PE among TPE

D-dimer	Sensitivity % (95% CI)	Specificity % (95% CI)	PPV % (95% CI)	NPV % (95% CI)
> 1.18 mg/L	89.7 (71.5–97.3)	77.8 (69.0–84.5)	49.1(35.3–63.0)	96.9 (90.6–99.2)

*PPV* positive predictive value, *NPV* negative predictive value

The PE patients had lower median WBC and IL-8 values (5.14 × 10^9^/L vs. 6.1 × 10^9^/L, *P* < 0.05; 30.2 pg/ml vs. 89.7 pg/ml, *P* < 0.05) but a higher median IL-2 receptor value (1964.8 pg/ml vs. 961.2 pg/ml, *P* < 0.01) than those in the non-PE patients. Other inflammatory biomarkers and cytokines, such as ESR, CRP, IL-6, and TNF-α, were not significantly different between the PE and non-PE groups (Table 3).

**Table 3:** Biochemical characteristics of patients with confirmed diagnosis (median, range)

	PE	Non-PE	*p* value
ESR (mm/h) (n = 150; 29/121)	54.5 (2–120)	44 (1–120)	0.127
CRP (mg/L) (n = 150; 29/121)	39.1 (1.39–200)	37 (2.46–196)	0.538
WBC (10^9^/L) (n = 150; 29/121)	5.14 (3.05–10.5)	6.1 (2.1–12.5)	0.022*
IL-8 (pg/ml) (n = 109; 22/87)	30.2 (7.97–58.1)	89.7 (5–354)	0.028*
IL-6 (pg/ml) (n = 109; 22/87)	22.3 (8.74–40)	21.5 (5–59.4)	0.487
IL-2 receptor (pg/ml) (n = 109; 22/87)	1964.8 (618–5444)	961.2 (463–5444)	0.005**
TNF-α (pg/ml) (n = 109; 22/87)	32.8 (10.9–66.5)	50.6 (7.52–201)	0.102

*p < 0.05, **p < 0.01

## Discussion

The current study investigated plasma D-dimer levels and their diagnostic performance in predicting PE in patients with TPE. Our results indicated that most (75%) patients with TPE had elevated D-dimer levels. Furthermore, D-dimer levels were significantly higher in PE patients than in non-PE patients in the TPE population. With a cut-off value of 1.18 mg/L, D-dimer showed a good diagnostic performance in predicting PE in TPE due to its high sensitivity (89.7%) and specificity (77.8). The NPV was 96.9% (95% CI 90.6–99.2), indicating an excellent rule-out ability at this cut-off value.

According to the patients included in our study, the most common symptoms of TPE were cough (74.6%), fever (51.7%), chest pain (50.8%), and dyspnoea (49.6%). Chest pain, haemoptysis, and dyspnoea consist of the PE triad without specificity [10]. When PE co-occurs with TPE, the symptoms are more complicated. It is difficult to distinguish PE patients from TPE patients based on their symptoms and signs alone. Furthermore, fever, chest pain, and dyspnoea often accelerate heart rate, which may increase the wells score [11]. Together with the elevated D-dimer levels, the clinical decision may be inclined to allow patients to undergo further radiological testing. Increased wells scores influenced by complications may partly explain the limited diagnostic accuracy of the C-PTP/D-dimer strategy for PE in patients with complications.

As reported above, D-dimer levels are elevated in most patients with TPE. Using a threshold of 0.5 mg/L in predicting and discriminating PE from non-PE in the TPE population was ineffective as specificities (nearly 0% according to our study) were extremely low. To reduce unnecessary examinations, an optimal cut-off for predicting PE in TPE is needed. According to the receiver operating characteristic curve analysis, the area under the curve of D-dimer was 0.893 (95% CI 0.839–0.947; *P* < 0.01), indicating a significant statistical correlation between D-dimer and PE among TPE patients. The cut-off point was determined as the value of the parameter that maximised the sum of specificity and sensitivity based on Youden’s index. D-dimer has a good diagnostic performance in predicting PE in TPE patients at the level of 1.18 for its high sensitivity (89.7%) and specificity (77.8%). According to our study, D-dimer had a low PPV of 49.1% (95% CI 35.3–63) but a high NPV of 96.9% (95% CI 90.6–99.2) when the cut-off value is set at 1.18 mg/L, which indicated an excellent rule out ability. PPV is correlated with the prevalence of disease and has limited predictive value in diseases with low prevalence [12]. The low PPV in our study may be partly due to the relatively low prevalence of PE.

According to this study, the morbidity of PE in TPE patients is no less than 11.7%, which is higher than that in the normal population [13]. To elucidate the underlying mechanism, we compared inflammatory biomarkers between PE and non-PE patients. The inflammatory processes of TPE are orchestrated by cytokines and chemokines, which also participate in all stages of embolism from early endothelial dysfunction to the late formation of embolus [14]. Maria et al. reviewed recent studies and revealed that cytokines, including interferon-γ, IL-6, CCL2, IL-17A, IL-9, IL-1β, and transforming growth factor-β, exert prothrombotic effects, while other cytokines, such as IL-10, tumour necrosis factor (TNF)-α, and IL-8, appear, to promote thrombus resolution in the late phase of venous thromboembolism [15]. In addition, another study revealed that IL-8 enhances thrombus resolution through neovascularization and neutrophil recruitment [16]. The limitation of these studies is that most of the subjects were experimental models. Studies on humans are scarce. In the current study, IL-8 levels and WBC counts of PE patients were significantly lower than those of non-PE patients, indicating dysfunction of antithrombotic cytokines in PE patients. IL-2 receptor levels were significantly higher in patients with PE than in those without. This result was consistent with the study of Mirjana that revealed a positive correlation between IL-2R and anti-annexin A5 antibodies, a risk factor for embolism, in patients with primary antiphospholipid syndrome and PE [17]. Nonetheless, IL-6, TNF-α, ESR, and CRP showed no significant differences between PE and non-PE patients in this study. Interestingly, none of the patients in this study who underwent deep vein ultrasound of the lower extremity had deep venous thromboembolism. Whether TPE promotes local venous thrombosis is worth further study. Our study sheds light on the role of cytokines in PE in patients with TPE. Prospective large-scale studies are needed to determine whether there is cytokine-induced procoagulant and anticoagulant dysfunction in patients with TPE.

There are several limitations to our study that must be addressed. First, the small number of patients must be emphasised, bearing the risk of sample size error. Second, our D-dimer cut-off level of 0.5 mg/L was not age-adjusted; for the case collection time span from 2014 to 2020, most of the early cases underwent CTPA when D-dimer was more than 0.5 mg/L. Third, the retrospective design of the study limited the richness of the research. Neither was the information associated with clinical pretest probability recorded nor could we study the diagnostic performance of C-PTP/D-dimer strategies in predicting PE among TPE patients. A prospective, ideally designed study with physicians randomly blinded to routinely collected D-dimers would be required to solve the above problems.

## Conclusions

Most patients with TPE had elevated plasma D-dimer levels. Among TPE patients, plasma D-dimer levels were higher in PE patients than in non-PE patients. To avoid unnecessary radiological tests, we should set the rule out cut-off point of D-dimer at 1.18 mg/L rather than a fixed 0.5 mg/L or age-adjusted D-dimer level in the TPE population. The imbalance of prothrombotic and antithrombotic cytokines may partly be attributed to the formation of pulmonary emboli in patients with TPE.

## Data Availability

The datasets used and/or analysed during the current study are available from Xiaoming Li (email: lixiaoming13734@126.com) on reasonable request.

## Abbreviations

CRP: C-reactive protein
CTPA: Computed tomography pulmonary angiography
C-PTP: Clinical pretest probability
ESR: Erythrocyte sedimentation rate
PE: Pulmonary embolism
ROC: Receiver operating characteristic
TB: Tuberculosis
TPE: Tuberculous pleural effusion
WBC: White blood cell
